# A systematic review and meta‐analysis of premature mortality in bipolar affective disorder

**DOI:** 10.1111/acps.12408

**Published:** 2015-03-03

**Authors:** J. F. Hayes, J. Miles, K. Walters, M. King, D. P. J. Osborn

**Affiliations:** ^1^ Division of PsychiatryUCLLondonUK; ^2^ Camden & Islington NHS Foundation TrustNHSLondonUK; ^3^ Department of Primary Care and Population HealthUCLLondonUK

**Keywords:** bipolar disorder, mortality, life expectancy

## Abstract

**Objective:**

To review and complete meta‐analysis of studies estimating standardised mortality ratios (SMRs) in bipolar affective disorder (BPAD) for all‐cause and cause‐specific mortalities.

**Method:**

Cause‐specific mortality was grouped into natural and unnatural causes. These subgroups were further divided into circulatory, respiratory, neoplastic and infectious causes, and suicide and other violent deaths. Summary SMRs were calculated using random‐effects meta‐analysis. Heterogeneity was examined via subgroup analysis and meta‐regression.

**Results:**

Systematic searching found 31 studies meeting inclusion criteria. Summary SMR for all‐cause mortality = 2.05 (95% CI 1.89–2.23), but heterogeneity was high (*I* ^2^ = 96.2%). This heterogeneity could not be accounted for by date of publication, cohort size, mid‐decade of data collection, population type or geographical region. Unnatural death summary SMR = 7.42 (95% CI 6.43–8.55) and natural death = 1.64 (95% CI 1.47–1.83). Specifically, suicide SMR = 14.44 (95% CI 12.43–16.78), other violent death SMR = 3.68 (95% CI 2.77–4.90), deaths from circulatory disease = 1.73 (95% CI 1.54–1.94), respiratory disease = 2.92 (95% CI 2.00–4.23), infection = 2.25 (95% CI 1.70–3.00) and neoplasm = 1.14 (95% CI 1.10–1.21).

**Conclusion:**

Despite considerable heterogeneity, all summary SMR estimates and a large majority of individual studies showed elevated mortality in BPAD compared to the general population. This was true for all causes of mortality studied.

**Summations**

- All‐cause and cause‐specific mortalities are elevated in individuals with bipolar affective disorder relative to the general population.
- As well as dramatic elevations in suicide risk, there is a mortality gap from all medical illnesses examined.

**Considerations**

- Heterogeneity was high in all‐cause and in many cause‐specific standardised mortality ratio estimates.
- Heterogeneity could not be accounted for by any of the study‐level variables available.

## Introduction

An increasing body of research has shown that bipolar affective disorder (BPAD) is associated with premature mortality. Where previously it was believed this was mostly attributable to unnatural causes such as suicide, homicide and accidents, it has also been shown that patients with BPAD are also at risk of premature death from a range of medical illnesses 1. In 1998, Harris and Barraclough 2 reviewed mortality in all mental disorders. Six studies contributed to their meta‐analysis of mortality in BPAD. A more recent review published in 2009 included 13 studies 1. However, this review only searched one database and included patients without a clear diagnosis of BPAD (such as mixed unipolar/bipolar groups). Since these publications, a number of large database studies have derived standardised mortality ratio (SMR) estimates. The SMR is an indirect method of standardisation calculated by the ratio of observed deaths in the study group to expected deaths in the general population.

The issue of premature mortality, beyond suicide, has been understudied in BPAD compared with other disorders, such as schizophrenia and unipolar depression 3, and there is a lack of clear evidence in this area. There is likely to be considerable heterogeneity amongst SMR estimates. Overreliance on either inpatient data or community‐based samples is an important limitation. The sole use of inpatient data may potentially result in bias and poor generalisability by including only more severe cases, whereas community‐based samples are often limited by insufficient sample sizes. Heterogeneity may also be introduced by period effects and by comparing treated and untreated groups.

### Aims of the study

To estimate all‐cause and cause‐specific mortalities in bipolar affective disorder via a systematic review and meta‐analysis of cohort studies.

## Material and methods

Existing studies of SMR in BPAD were systematically reviewed to examine the association between BPAD and all‐cause and cause‐specific mortality. Cause‐specific mortality was grouped into natural and unnatural causes. These subgroups were further divided into suicide and other violent deaths, and deaths from circulatory, respiratory, neoplastic and infectious causes. Heterogeneity was assessed by geographical region, population type, cohort size, mid‐decade of cohort data collection and decade of publication. We closely followed the guidance provided by the PRISMA statement and MOOSE proposal for reporting 4, 5.

### Identification of studies

**Figure 1:**
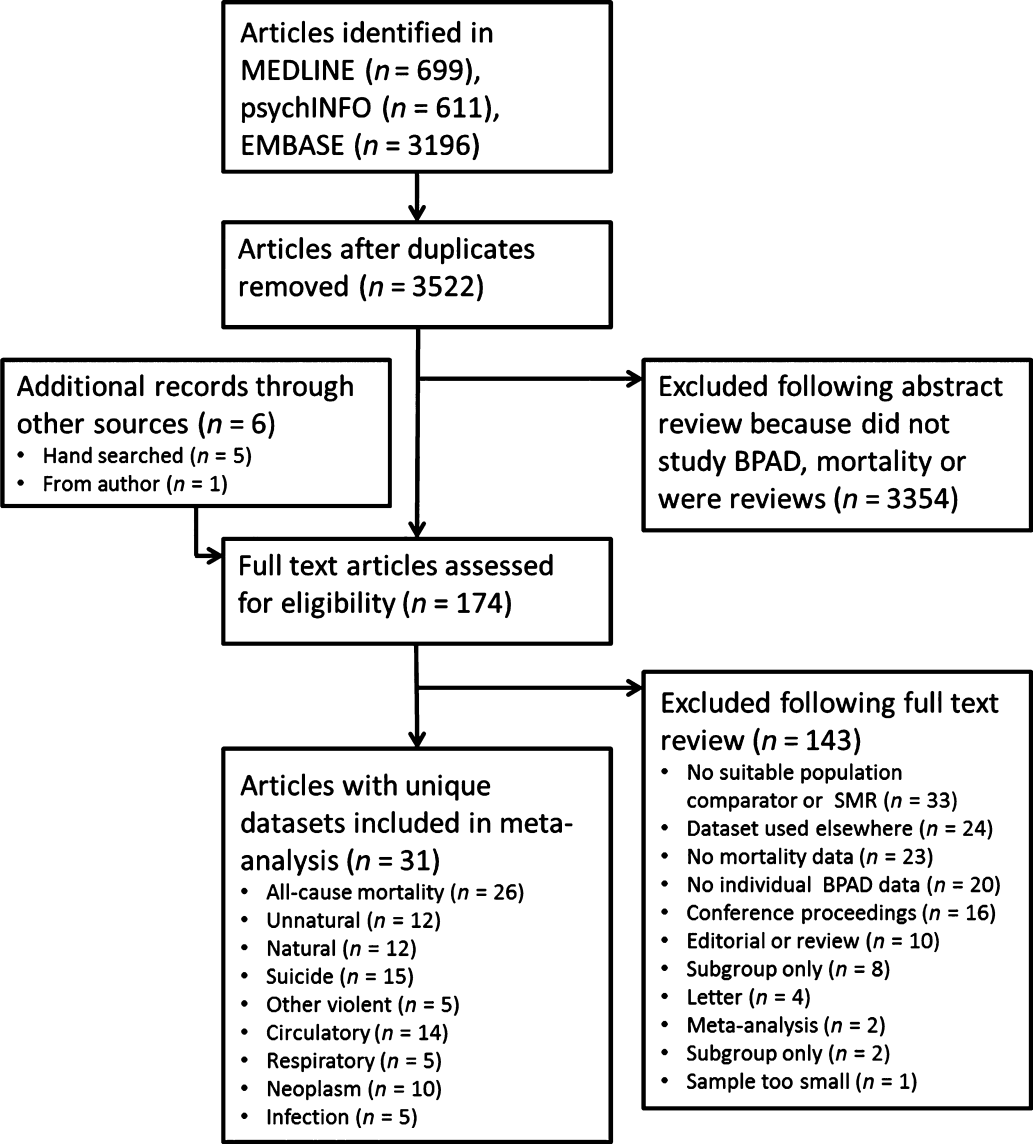
Flow diagram of the published articles evaluated for inclusion in this meta‐analysis.

To identify all studies examining mortality in BPAD, the Medical Subject Heading (MeSH) terms and keywords for BPAD and mortality were searched in PsycINFO, MEDLINE and EMBASE. MeSH terms were as follows: *bipolar disorder*,* mortality*,* life expectancy*,* death*,* death and dying*. Keywords searched were as follows: *bipolar illness*,* manic depression*,* bipolar disorder*,* bipolar affective disorder*,* life expectancy*,* mortality and death* (full search terms are available from the corresponding author on request). All databases were searched from their inception until 30 July 2014. JFH and JM performed the searches individually and then compared results. The abstracts of potentially relevant articles were reviewed by both JFH and JM. Additional articles and conference papers including primary data were identified from citations in relevant studies and reviews, the Cochrane database of systematic reviews and Google Scholar. Emails were then sent to senior authors of articles that met inclusion criteria to attempt to identify all missing studies. One extra‐published study was identified by this method, and no further unpublished data were made available (Fig. 1).

### Inclusion and exclusion criteria

Included studies met all of the following *a priori* defined criteria:

- Published between 1 January 1960 and 30 July 2014.
- Reported deaths of people diagnosed with BPAD; studies were included if BPAD was diagnosed by any criteria.
- Individuals included in the study were 16 years or older.
- Primary data on all‐cause mortality or cause‐specific mortality were included; specific subcategories of mortality were as follows: natural deaths, unnatural deaths, suicide, other violent deaths, infection, neoplasm, respiratory and circulatory system disease.
- Reported data on observed and expected deaths, or SMR allowing the number of observed and expected deaths to be calculated.

Studies were excluded if they:

- Involved cohorts that could not be defined as having BPAD (i.e. studies which grouped together affective disorders).
- Included a cohort of <50 patients (to avoid including cohorts in which there were no observed deaths).
- Were not standardised by age.
- Reported mortality in a particular subgroup of the population with BPAD (i.e. prison population).
- Reported duplicate data (or data sets from overlapping time periods at the same site).

Study‐level information was filtered to identify data from multiple studies that overlapped in place and time. The most informative paper was then used as the representative mortality estimate for inclusion in the meta‐analysis (i.e. larger samples and longer time periods were preferred).

### Data extraction

Once a study was included, data were extracted and entered into a database that included study‐level variables [authors, country, year of publication, years of data collection, length follow‐up, which covariates the mortality was standardised by, the site of collection (i.e. multiple site or population level), the population type (i.e. inpatient or community)] and estimate‐level variables (number of men and women in the cohort, deaths from all causes and specific causes for both men and women, and population‐level estimates of expected deaths).

JFH and JM individually extracted data used in the analysis using a standardised form. If disagreements arose, these were resolved by consensus. When required, we contacted the original authors for clarification of issues.

### Statistical methods

The SMR gives the ratio of death in BPAD compared to the general population. For each cause of death, SMRs and their 95% confidence intervals were extracted from each publication or calculated (observed deaths/expected deaths).

The statistical significance of the SMR is based on the Poisson distribution (two‐tailed) using 95% confidence intervals. The SMR is significantly raised when the lower confidence interval is >1.00. For each study, 95% CIs were calculated using the Rothman–Greenland method 6. Pooled SMRs with 95% CI for all‐cause and cause‐specific mortalities were calculated using the DerSimonian and Laird method, a random‐effects model that incorporates both between‐study and within‐study variation 7. Using this method assumes that significant heterogeneity exists between studies.

Statistical heterogeneity was assessed in a number of ways. First, the *I* ^2^ index and chi‐square test were used to investigate differences amongst studies with respect to SMRs. Additionally, meta‐regression analysis was performed for heterogeneity of the all‐cause SMR because of decade of publication, cohort size, geographical region, mid‐decade of cohort data collection and population type (i.e. inpatient or community). Subgroup analyses were performed to assess potential sources of heterogeneity separately as a result of the following available patient‐level and study‐level factors: geographical region of study, patient population type and decade of the middle year of patient observation. These were considered to be the key sources of potential bias in the included studies. Funnel plots and Egger's regression were used to assess for publication and small‐study bias in groups containing 10 or more studies 8.

All analysis was completed using metan and associated commands in stata 13 9.

## Results

The inclusion criteria were met by 31 published studies including unique data sets 10, 11, 12, 13, 14, 15, 16, 17, 18, 19, 20, 21, 22, 23, 24, 25, 26, 27, 28, 29, 30, 31, 32, 33, 34, 35, 36, 37, 38, 39, 40 (Fig. 1, Table 1). Of these, 64% were inpatient cohorts. A large number (45%) of studies came from Scandinavian countries (Norway, Sweden, Denmark and Finland). Overall, there were 305 859 people with a diagnosis of BPAD (not including individuals in studies 24 and 31, which only presented person‐years at risk). Data collection ranged from 1935 to 2010.

**Table 1:** Studies included in the meta‐analysis

Author (year)	Country	Years of collection	Total, *N*	*N*, Men	*N*, Women	Standardised by	Site of collection	Population type	Mortality outcome
Bratfos & Haug (1968) (10)	Norway	1950–1963	207			Age, Sex	Single site	Inpatient	All‐cause, suicide
Innes & Miller (1970) (11)	United Kingdom	1964–1969	374			Age	Population	Inpatient & community	All‐cause
Kay & Petterson (1977) (12)	Sweden	1961–1970	192	84	108	Age, sex	Multiple site	Inpatient	All‐cause
Tsuang et al. (1980) (13)	United States	1935–1974	100	45	55	Age, sex	Single site	Inpatient	All‐cause, unnatural, natural, infectious , neoplasm, circulatory
Norton et al. (1984) (14)	United Kingdom	1967–1976	791			Age, sex	Population	Inpatient & community	All‐cause, suicide, infectious, neoplasm, circulatory
Black et al. (1987) (15)	United States	1970–1981	586	266	320	Age, sex, time at risk	Single site	Inpatient	All‐cause, unnatural, natural
Weeke et al. (1987) (16)	Denmark	1969–1976	417	185	232	Age, sex, time at risk	Inception cohort	Inpatient	All‐casue, unnatural, natural, suicide, other violent, neoplasm, circulatory
Newman & Bland (1991) (17)	Canada	1976–1985	1429			Age, sex, time at risk	Multiple site	Inpatient & community	All‐cause, suicide
Vestergaard & Aagaard (1991) (18)	Denmark	1981–1988	133			Age, sex	Multiple site	Inpatient	All‐cause, suicide, circulatory, respiratory
Jorgensen & Mortensen (1992) (19)	Denmark	1970–1988	18293			Age, sex	Inception cohort	Inpatient	All‐cause
Sharma et al. (1994) (20)	United Kingdom	1970–1987	472			Age, sex	Population	Inpatient & community	All‐cause, suicide, circulatory, respiratory
Ahrens et al. (1995) (21)	Denmark, Germany, Canada, Austria	1962–1992	440	189	251	Age, sex	Multiple site	Inpatient & community	All‐cause, suicide, circulatory
Nilson (1995) (22)	Sweden	1970–1991	362			Age, sex	Single site	Inpatient	All‐cause
Saku et al. (1995) (23)	Japan	1948–1982	187	119	68	Age	Single site	Inpatient	All‐cause, neoplasm
Hiroeh et al. (2001) (24)	Denmark	1973–1993	N/A	N/A	N/A	Age, sex, time at risk	Population	Inpatient	Unnatural, suicide, other violent
Osby et al. (2001) (25)	Sweden	1973–1995	15386	6578	8808	Age, sex	Population	Inpatient	All‐cause, unnatural, natural, suicide, other violent, infectious, neoplasm, circulatory, respiratory
Schneider et al. (2001) (26)	Germany	1983–1988	74	24	50	Age, sex	Single site	Inpatient	All‐cause, unnatural
Angst et al. (2002) (27)	Switzerland	1959–1997	220			Age	Single site	Inpatient	All‐cause, unnatural, natural, suicide, other violent, neoplasm, circulatory
Amaddeo et al. (2007) (28)	Italy	1982–2001	278			Age, sex	Population	Community	All‐cause
Dutta et al. (2007) (29)	United Kingdom	1965–1999	135	102	133	Age, sex	Inception cohort	Community	All‐cause, natural, suicide, neoplasm
Osborn et al. (2007) (30)	United Kingdom	1987–2002	69056			Age, sex	Population	Community	Circulatory
Hiroeh et al. (2008) (31)	Denmark	1973–1993	N/A	N/A	N/A	Age, sex, time at risk	Population	Inpatient	Natural, infectious, neoplasm, circulatory, respiratory
Osborn et al. (2008) (32)	United Kingdom	1987–2002	10742			Age, sex	Population	Community	Suicide
Chang et al. (2010) (33)	United Kingdom	2007–2009	2700			Age, sex	Inception cohort	Community	All‐cause
Hoang et al. (2011) (34)	United Kingdom	1999–2006	75718			Age, sex	Population	Inpatient	All‐cause, unnatural, natural
Nordentoft et al. (2011) (35)	Denmark	1970–2006	5927	2571	3356	Age, sex	Inception cohort	Inpatient & community	Suicide
Ajetunmobi et al. (2013) (36)	United Kingdom	1986–2010	3839			Age, sex, deprivation, time at risk	Inception cohort	Inpatient	All‐cause, unnatural, natural, suicide, infectious, neoplasm, circulatory
Crump et al. (2013) (37)	Sweden	2003–2009	6618	2700	3918	Age, sex	Population	Inpatient & community	All‐cause, unnatural, natural, suicide, other violent, infectious, neoplasm, circulatory, respiratory
Hoang et al. (2013) (38)	United Kingdom	2006–2008	34707			Age, sex	Population	Inpatient	All‐cause
Laursen et al. (2013a) (39)	Sweden	2000–2007	18355	7367	10988	Age, sex	Population	Inpatient	All‐cause, unnatural, natural, circulatory
Laursen et al. (2013b) (39)	Finland	2000–2007	9919	4489	5430	Age, sex	Population	Inpatient	All‐cause, unnatural, natural, circulatory
Laursen et al. (2013c) (39)	Denmark	2000–2007	11101	4280	6821	Age, sex	Population	Inpatient	All‐cause, unnatural, natural, circulatory
Westman et al. (2013) (40)	Sweden	1987–2006	17101	8208	8893	Age, sex	Population	Inpatient	All‐cause, unnatural, natural, circulatory

N/A, not available

Hiroeh et al. (24 & 31) presented person‐years at risk (PYAR) rather than individuals: men = 155337 PYAR, women = 309639 PYAR.

**Figure 2:**
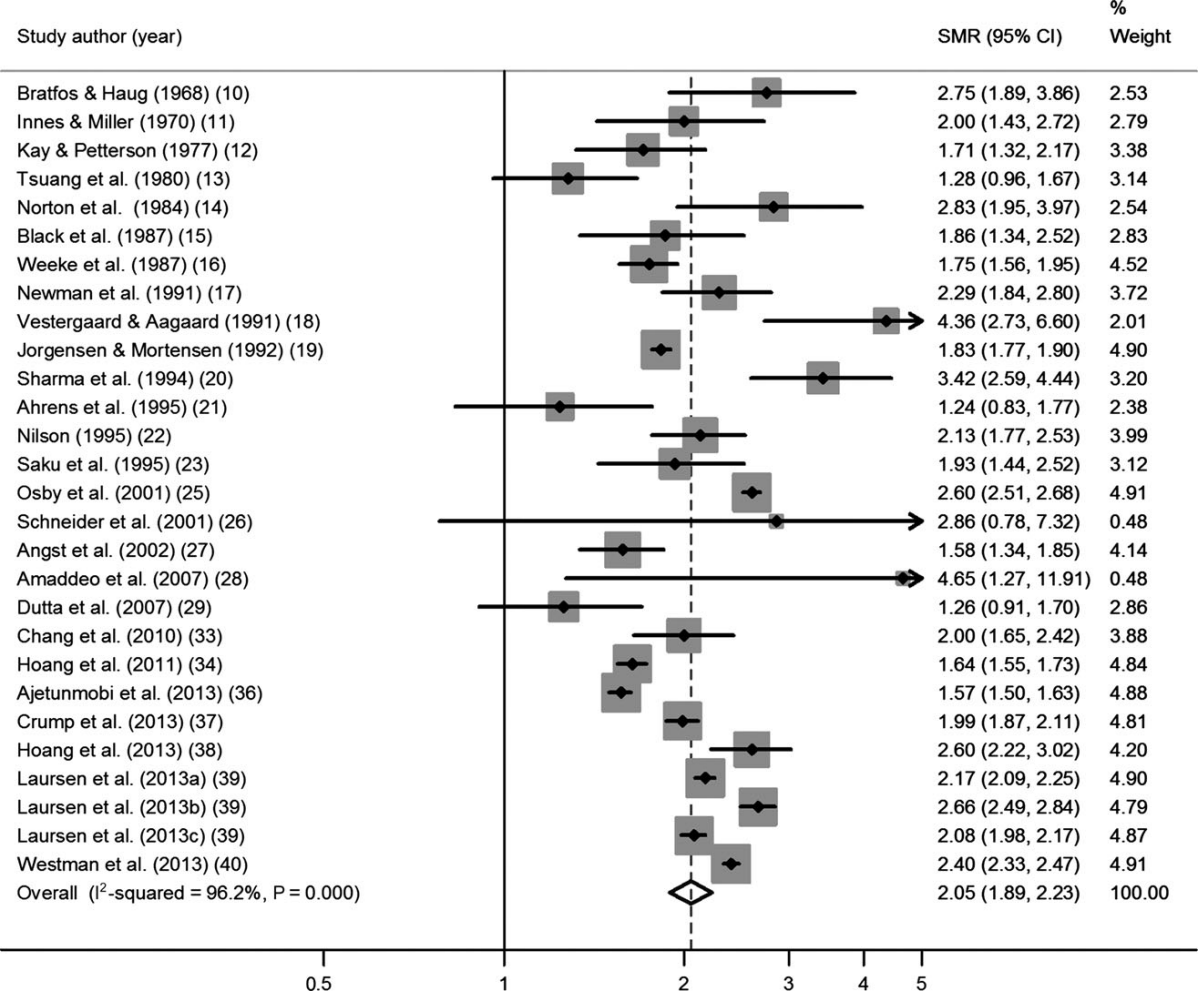
Standardised mortality ratio (SMR) of all‐cause mortality. CI, confidence interval; % weights are from random‐effects analysis.

The reported SMRs for all‐cause mortality in patients with BPAD ranged from 1.24 (95% CI 0.83–1.17) to 4.65 (95% CI 1.27–11.91). Within the 26 individual studies assessing all‐cause mortality, all SMR point estimates were elevated, but four of 26 had confidence intervals that overlapped one 13, 21, 26, 29, and these were all relatively small studies (*N* < 440). The all‐cause summary SMR for bipolar disorder was 2.05 (95% CI 1.89–2.23). There was significant heterogeneity between these studies (*I* ^2^ = 96.2%, 95% CI 95.6–96.7, *P* < 0.001) (Fig. 2).

Sex‐specific all‐cause mortality showed similarly elevated summary estimates, but again the studies were highly heterogeneous (Table 2). This was also true for mortality grouped as natural and unnatural. Studies had heterogeneous SMR estimates for suicide, other violent deaths and circulatory disease mortality. Estimates for SMRs for infectious and neoplastic deaths were more homogenous. Summary estimates suggested increased rates of death from all causes in people with bipolar disorder (Table 2).

**Table 2:** Summary SMRs for all‐cause and cause specific mortalities

	No. of studies	Number of individuals^a^	Summary SMR (95°% CI)	*I* ^2^ (95% CI)	Het *P*
All‐cause	26	220 134	2.05 (1.89–2.23)	96.2 (95.6–96.7)	<0.001
Men	15	34 636	2.17 (2.01–2.34)	83.6 (74.8–88.3)	<0.001
Women	15	46 075	2.11 (1.93–2.31)	84.4 (83.4–91.39)	<0.001
Natural	12	159 495	1.64 (1.47–1.83)	98.0 (98.0–98.5)	<0.001
Men	9	34 220	1.72 (1.54–1.93)	94.5 (92.5–95.8)	<0.001
Women	9	45 598	1.74 (1.51–1.99)	97.6 (97.1–98.0)	<0.001
Unnatural	12	159 434	7.42 (6.43–8.55)	95.6 (93.9–96.2)	<0.001
Men	9	34 142	7.89 (7.05–8.81)	82.4 (68.1–88.6)	<0.001
Women	9	45 515	9.23 (7.14–11.94)	96.6 (95.6–97.2)	<0.001
Suicide	15	46 756	14.44 (12.43–16.78)	87.0 (80.4–90.7)	<0.001
Men	9	12 325	13.31 (10.62–16.69)	87.8 (78.7–91.9)	<0.001
Women	9	16 698	15.74 (12.84–19.31)	81.7 (63.4–88.7)	<0.001
Other violent	5	22 641	3.68 (2.77–4.90)	89.5 (77.2–93.8)	<0.001
Men	4	9463	3.06 (2.19–4.03)	86.2 (57.6–92.8)	<0.001
Women	4	12 958	5.53 (1.60–19.14)	99.0 (98.7–99.19)	<0.001
Circulatory	14	153 948	1.73 (1.54–1.94)	95.2 (93.9–96.1)	<0.001
Men	9	34 041	1.81 (1.61–2.05)	90.3 (85.1–93.1)	<0.001
Women	9	45 396	1.72 (1.46–2.03)	96.0 (94.8–96.82)	<0.001
Respiratory	5	22 609	2.92 (2.00–4.23)	94.14 (89.8–96.1)	<0.001
Men	4	9278	2.73 (1.76–4.24)	90.2 (75.9–94.5)	<0.001
Women	4	12 726	2.72 (1.78–4.20)	91.3 (80.0–94.99)	<0.001
Infection	5	22 895	2.25 (1.70–3.00)	45.4 (0.0–78.5)	0.12
Men	4	9323	2.76 (1.94–3.92)	43.1 (0.0–80.0)	0.15
Women	4	12 781	1.77 (1.30–2.40)	20.9 (0.0–74.1)	0.29
Neoplasm	10	27 693	1.14 (1.10–1.21)	20.7 (0.0–61.9)	0.25
Men	7	9729	1.11 (1.05–1.17)	0.0 (0.0–58.5)	0.47
Women	7	13 214	1.19 (1.05–1.37)	56.3 (0.0–79.3)	0.03

SMR, standardised mortality ratio; CI, confidence interval; *I* ^2^, index of heterogeneity; Het *P*, from *x* ^2^ test.

a Not including individuals in studies 24 and 31.

In univariable meta‐regression, all‐cause mortality was not significantly associated with decade of publication (*P* = 0.63), cohort size (*P* = 0.75), geographical region (*P* = 0.55), mid‐decade of cohort data collection (*P* = 0.89) or population type (*P* = 0.65). After accounting for all of these possible explanatory variables in multivariable meta‐regression, residual variation due to heterogeneity amongst all‐cause mortality SMRs remained (*I* ^2^ = 88.3%, 95% CI 84.6–90.7, *P* < 0.001).

Subgroup analyses were performed stratified by geographical region, population type and mid‐decade of study (Table 3). Stratifying by these covariates had little effect on heterogeneity in summary estimates for all‐cause, natural and unnatural death SMRs, which remained high.

**Table 3:** Summary SMRs by subgroup for all‐cause mortality, and natural and unnatural death

	No. of studies	Summary SMR (95% CI)	*I* ^2^ (95% CI)	Het *P*
All‐cause				
Geographical region				
Scandinavia	10	2.20 (2.01–2.41)	96.5 (95.2–97.5)	<0.001
North America	3	1.77 (1.24–2.53)	81.4 (42.7–94.0)	0.005
UK	8	2.06 (1.74–2.34)	91.7 (86.0–95.1)	<0.001
Other European	4	1.67 (1.18–2.38)	51.7 (0.0–84.1)	0.1
Japan	1	1.93 (1.44–2.52)	–	–
Population type				
Inpatient	17	2.05 (1.86–2.25)	97.3 (96.9–97.7)	<0.001
Inpatient and community	6	2.21 (1.79–2.73)	80.6 (49.1–89.4)	<0.001
Community	3	1.85 (1.16–2.94)	77.6 (0.0–91.1)	0.01
Mid‐point of study				
1950s	2	1.86 (0.88–3.94	90.9^a^	0.001
1960s	3	1.85 (1.58–2.17)	0.0 (0.0–72.9)	0.7
1970s	6	1.93 (1.67–2.24)	82.5 (60.8–89.8)	<0.001
1980s	6	2.30 (1.84–2.87)	84.1 (62.3–90.9)	<0.001
1990s	3	2.12 (1.42–3.14)	99.3 (99.0–99.4)	<0.001
2000s	5	2.13 (1.90–2.39)	95.7 (93.9–96.8)	<0.001
Natural				
Geographical region				
Scandinavia	6	1.79 (1.56–2.05)	98.8 (98.6–99.0)	<0.001
North America	2	1.34 (0.90–2.00)	64.9^a^	0.09
UK	3	1.42 (0.95–2.12)	98.7 (98.1–99.0)	<0.001
Other European	1	1.40 (1.17–1.66)	–	–
Population type				
Inpatient	10	1.67 (1.48–1.88)	96.1 (95.0–96.9)	<0.001
Inpatient and community	1	1.79 (1.68–1.91)	–	–
Community	1	1.03 (0.71–1.44)	–	–
Mid‐point of study				
1950s	1	1.10 (0.79–1.49)	–	–
1960s	0	–	–	–
1970s	3	1.36 (1.23–1.51)	0.0 (0.0–72.9)	0.43
1980s	3	1.51 (1.14–2.00)	99.1 (98.8–99.3)	<0.001
1990s	2	2.06 (2.00–2.12)	0.0^a^	0.62
2000s	3	1.75 (1.50–2.03)	97.1 (95.8–97.9)	<0.001
Unnatural				
Geographical region				
Scandinavia	6	8.15 (7.20–9.24)	94.0	<0.001
North America	2	3.12 (1.97–4.96)	0^a^	0.75
UK	2	7.90 (3.16–19.73)	99.2^a^	<0.001
Other European	2	5.60 (2.42–12.95)	31.8^a^	0.23
Population type				
Inpatient	11	7.56 (6.52–8.77)	95.4 (94.0–96.3)	<0.001
Inpatient and community	1	6.05 (5.14–7.12)	–	–
Community	0	–	–	
Mid‐point of study				
1950s	1	2.93 (1.52–5.13)	–	–
1960s	0	–	–	–
1970s	3	5.46 (3.03–9.85)	84.3 (15.7–93.0)	0.002
1980s	3	8.75 (6.39–11.97)	97.3 (95.4–982)	<0.001
1990s	2	6.93 (3.60–13.34)	98.9^a^	<0.001
2000s	3	8.26 (6.53–10.44)	94.9 (91.6–96.6)	<0.001

SMR, standardised mortality ratio; CI, confidence interval: *I* ^2^, index of heterogeneity; Het *P*, from chi‐square test.

a 95% CI cannot be calculated due to 1 degree of freedom.

For all‐cause mortality SMR, Eggers test did not suggest significant publication bias (*P* = 0.63). The same was true for SMR of unnatural deaths (*P* = 0.55) and suicide (*P* = 0.40). However, given the high heterogeneity publication bias cannot be ruled out with certainty in these groups. It is even more likely to be present in studies of natural deaths (*P* = 0.05) and circulatory disease (*P* = 0.17).

## Discussion

This review of mortality in patients with BPAD highlights the increased risk of death from all causes. Summary SMR estimates from random‐effects meta‐analysis showed that all‐cause mortality in BPAD is double that expected in the general population. Natural deaths are over 1.5 times greater in BPAD than the general population; these natural deaths are made up of an almost double risk of deaths from circulatory illnesses (heart attacks, strokes, etc.) and three times risk of deaths from respiratory illness (COPD, asthma, etc.). Unnatural deaths are around seven times more common, with increased risk of suicide of around 14 times and other violent deaths (accidents, homicide, etc.) almost four times as likely. Deaths by all causes were similarly elevated in both men and women. Of particular concern is the lack of association between mid‐decade of cohort follow‐up and SMR: having BPAD in the 2000s has the same mortality risk compared to the general population as it did in the 1950s. With the increased use of second‐generation antipsychotics in these cohorts 41, 42 and associated elevated risk of cardiovascular disease, the failure of smoking cessation policy to address the needs of the severely mentally ill, relative to the general population 43, 44 and the continued lack of equality in access to health care for people with BPAD 43, this gap in deaths from medical illness may to widen unless it is directly addressed. In terms of modifying the increased rate of unnatural deaths, particular attention needs to be paid to comorbid substance misuse, risk of coercion, exploitation, receipt and perpetration of violence, and suicidal ideation 43, 44, 45.

Heterogeneity across studies was high (less so for deaths from cancer and infection). Heterogeneity in all‐cause mortality, and natural and unnatural death SMRs could not be accounted for by year of publication, study size, mid‐point of data collection, geographical region or population type. Whilst it is possible that some of these factors were imperfectly adjusted for in the analysis (for example some cohorts spanned many decades), the results suggest that there are unidentified factors that led to differences in outcomes for different cohorts of patients with BPAD. It has been shown that patients with BPAD in the United States have worse physical health and greater comorbidity than those in Germany and the Netherlands 47; however, it may be the case that there are even more localised differences in BPAD outcomes. This meta‐analysis suggests that within the US or Europe, mortality estimates for BPAD are not homogenous. Adjusting for cohort size did little to reduce the heterogeneity, despite the increased accuracy in SMR estimates larger studies should provide. Stratifying by decade of data collection did not affect heterogeneity, suggesting that the differences are not down to improvements in treatment over time. Studies of inpatient populations and community cohorts were also heterogeneous, suggesting these differences are not down to engagement with services or severity of illness.

There are several limitations of studies included in this review. SMRs were often only age and sex adjusted; therefore, other characteristics of the study populations, such as illness duration and lifestyle factors, may have contributed to the significant heterogeneity. For example, it was not possible to assess whether current or former smoking contributed to excess respiratory mortality. Disease severity was not assessed in all of the included studies, and therefore, it is not possible to assess heterogeneity in the overall mortality by severity. It has been recognised that patients with bipolar disorder accumulate numerous medical risk factors including smoking, use of alcohol and other illicit drugs, prescribed medication and comorbid anxiety and eating disorders that lead to earlier disease onset, poor engagement with health care and poor long‐term outcomes 48. These risk factors may be assigned differentially, both geographically and temporally. In many of the included studies, cause of death was ascertained from death certificates and therefore may be subject to potential misclassification bias. Having a mental health diagnosis has been shown to increase the risk of a coroners verdict of suicide rather than accidental or undetermined death 49 but may also reduce diagnosis of terminal illness leading to miscoding of physical cause of death 43.

This meta‐analysis highlights differential mortality in patients with BPAD and the general population. Similarly to schizophrenia, patients with BPAD have over twice the all‐cause mortality 50. Mortality from all physical conditions and unnatural causes is elevated. Variation in all‐cause mortality is considerable across time and place. There is no evidence that all‐cause mortality for patients with BPAD has improved over time relative to the general population.

## Declaration of interest

None.
